# Laparoscopic gastrectomy with lymph node dissection for the treatment of remnant stomach gastrointestinal stromal tumors in incomplete-type Carney’s triad: a case report

**DOI:** 10.1186/s40792-020-00877-y

**Published:** 2020-05-24

**Authors:** Yuhi Shimura, Shingo Kanaji, Naoki Urakawa, Masashi Yamamoto, Masako Utsumi, Gousuke Takiguchi, Hiroshi Hasegawa, Yoshiko Matsuda, Kimihiro Yamashita, Takeru Matsuda, Taro Oshikiri, Tetsu Nakamura, Satoshi Suzuki, Yoshihiro Kakeji

**Affiliations:** Division of Gastrointestinal Surgery, Department of Surgery, Graduate School of Medicine, Kobe University, 7-5-2 Kobeshi, Chuou-ku, Kusunoki-cho, Kobe, Hyogo 650-0017 Japan

**Keywords:** Carney’s triad, Gastrointestinal stromal tumors, Succinate dehydrogenase tumor deficiency

## Abstract

**Background:**

We report a rare case of gastrointestinal stromal tumors (GISTs) in Carney’s triad, successfully treated using laparoscopic gastrectomy with lymph node dissection after chemotherapy.

**Case presentation:**

A 21-year-old woman presented to our hospital for treatment of recurrent GISTs. The patient had been admitted for treatment 11 years prior, with black stools being the chief presenting complaint at that time. On examination at that time, multiple submucosal tumors in the pyloric antrum and multiple pulmonary tumors had been observed. She underwent open partial gastrectomy, and the diagnosis of GISTs was confirmed. She was administered tyrosine kinase inhibitors to treat lung metastases from 2 months after surgery. Due to the increasing size of the lung tumors, a right upper lobectomy was performed 9 years after the index gastric surgery. Histopathological examination of the lung specimen, in combination with re-examination of the gastric specimens, was indicative of incomplete-type Carney’s triad. Eleven years after the index gastric surgery, multiple GISTs were observed in her entire stomach. Tumor biopsy revealed a succinate dehydrogenase deficiency, confirming the diagnosis of recurrent GISTs. For treatment, the patient underwent laparoscopic completion gastrectomy, with D1 plus lymph node dissection.

**Conclusion:**

This is a first case report of completion gastrectomy performed laparoscopically for the treatment of GISTs associated with incomplete-type Carney’s triad. The recurrent GISTs developed over a protracted period of 11 years from the index gastric surgery to tumor recurrence.

## Background

In 1977, Carney et al. reported on the association between gastrointestinal stromal tumors (GISTs) and the presence of functioning extra-adrenal paraganglioma and pulmonary chondroma [1]. The presence of these three types of tumors together is now known as Carney’s triad [1]. When only two of these three tumor types are present, a diagnosis of incomplete-type Carney’s triad is applied [1, 2].

Generally, the medical course of Carney’s triad is protracted, with multiple tumor recurrence [3, 4]. One study reported a mean latency between the first tumor detection and tumor recurrence of about 8 years [3]. As it is known that tumors associated with Carney’s triad are resistant to tyrosine kinase inhibitors (TKIs) [5], surgical resection is the only curative treatment for GISTs resulting from Carney’s triad [6]. Partial resection is initially indicated if a single GIST is the primary lesion [3, 7, 8]. Although previous studies have reported on the surgical management of GISTs in primary Carney’s triad, the use of a laparoscopic approach to perform a completion gastrectomy for the treatment for this type of GIST has not been described previously.

## Case presentation

The patient was a 21-year-old woman, with an unremarkable family medical history. She had first undergone treatment for GISTs associated with an incomplete-type Carney’s triad 11 years prior, at the age of 10 years.

**Fig. 1:**
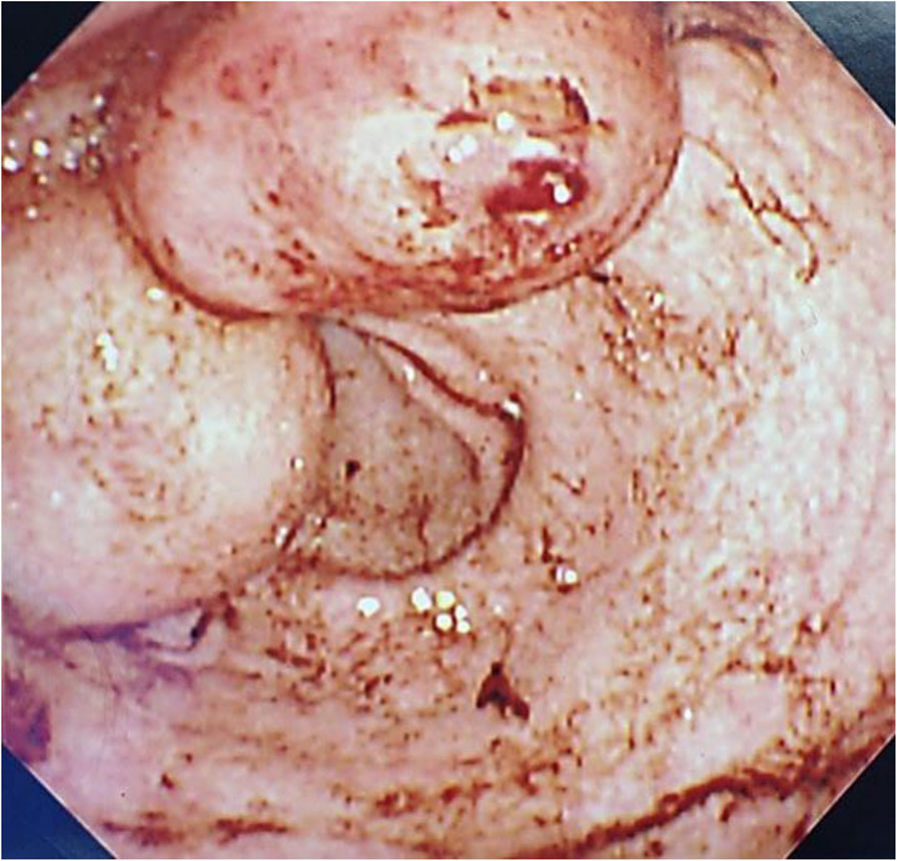
Endoscopic examination of the upper gastrointestinal tract showing the presence of two submucosal tumors in the pyloric antrum

**Fig. 2:**
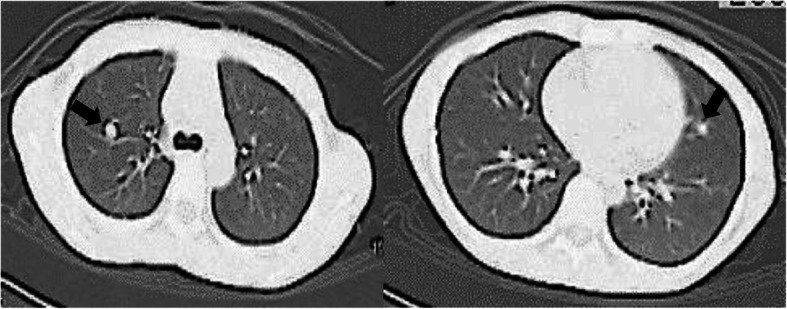
Chest plain computed tomography scan showed pulmonary nodular shadows of the S1 region in the right lung and lingular region in the left lung

**Fig. 3:**
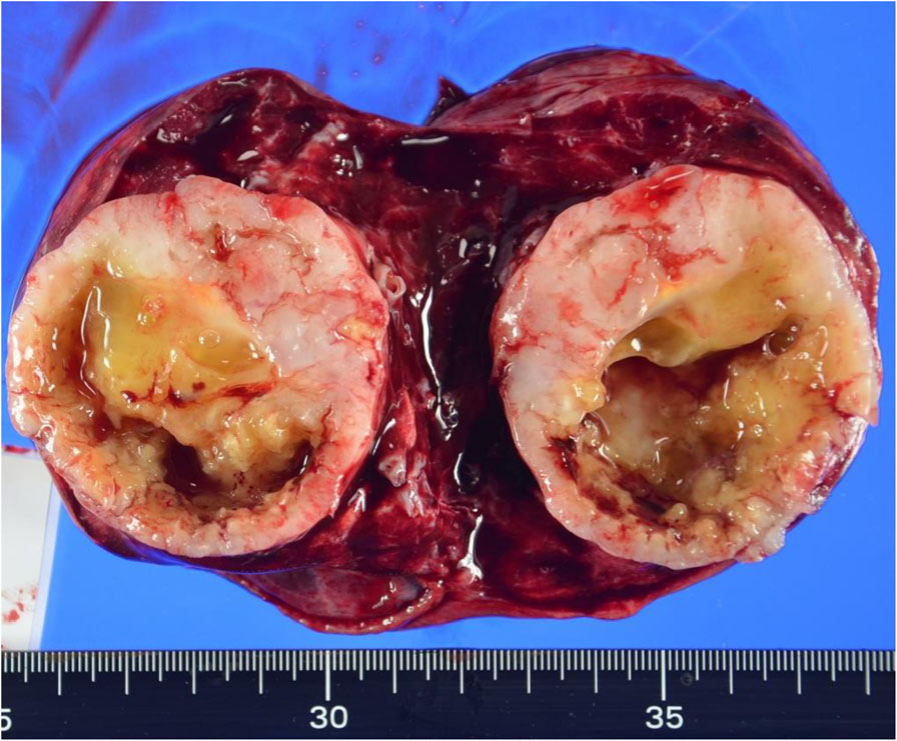
The divided surface of resected specimen of the lung. The pulmonary chondroma was a white lesion and was easily separated from surrounding lung tissue

At the time of her first admission and treatment, the patient had presented with black stools as her chief complaint. Endoscopic examination revealed the presence of two submucosal tumors in the pyloric antrum (Fig. 1) [9]. Magnetic resonance imaging showed multiple nodular tumors presenting as low signals on the T1-weighted image and high signals on T2-weighted image of the gastric body to antrum. In addition, computed tomography showed pulmonary nodular shadows in the S1 region in the right lung and lingular region in the left lung (Fig. 2) [9]. Based on these findings, the patient underwent open partial gastrectomy with Billroth-I reconstruction. Histopathological examination showed a positive result for c-kit and negative for S-100 protein; thus, we provided a diagnosis of wild-type GISTs. There was no vessel or lymphatic invasion, and MIB-1 labeling index was below 10%. The GISTs were diagnosed to be of high risk because the pulmonary tumors were considered to be metastatic. Two months after this gastric surgery, imatinib was initiated for the treatment of the pulmonary tumors. As the pulmonary tumors grew slowly, the type of TKI was changed from imatinib to a combination of sunitinib and regorafenib. This change did not lead to a positive tumor response. As the pulmonary tumors in the right upper lobe increased in size, a right upper lobectomy was performed, 9 years after the index gastric surgery. The tumor was uncoated and displaced the surrounding lung tissue (Fig. 3) [9]. Histopathological examination of the resected tumors confirmed the diagnosis of pulmonary chondroma. There was no vessel or lymphatic invasion. Based on the pathological result of pulmonary chondroma, a further histopathological re-evaluation of the resected gastric specimen was conducted that revealed a succinate dehydrogenase (SDH) deficiency of the tumors. Based on these findings, a diagnosis of an incomplete-type Carney’s triad was provided. The patient was followed up with yearly ultrasound examinations, with no indication of recurrent tumors in her stomach over the short-term follow-up.

**Fig. 4:**
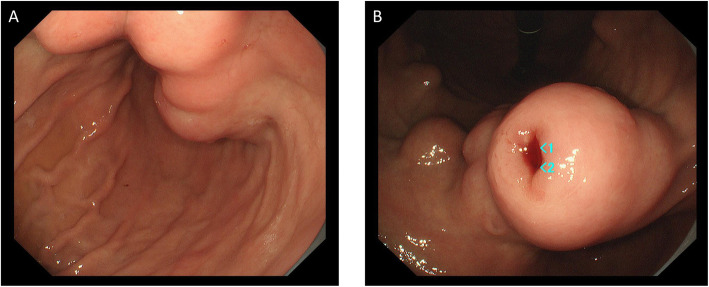
**a** Endoscopic examination of the upper gastrointestinal tract showing the presence of multiple submucosal tumors on the residual side of the lesser curvature. **b** Biopsy of the tumor confirmed the pathological diagnosis of GIST

**Fig. 5:**
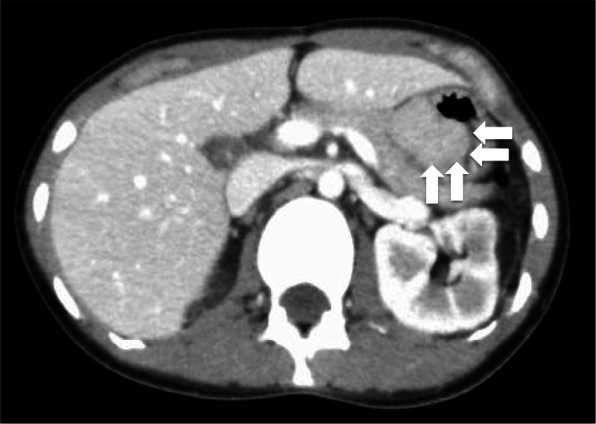
Computer tomography image showing multiple gastric masses in the entire stomach, growing inwards. There was no obvious disseminated involvement nor metastatic lesion

However, 2 years after her right upper lobectomy, at the age of 21 years, new gastric tumors were observed with ultrasound examination. Endoscopic examination of the upper gastrointestinal tract revealed the presence of multiple submucosal tumors on the residual side of the lesser curvature (Fig. 4a, b). Biopsy confirmed a pathological diagnosis of GISTs, with SDH deficiency. On computed tomography imaging, multiple masses were observed in the whole stomach, growing into the inner cavity. There was no obvious disseminated involvement nor metastatic lesions (Fig. 5). The patient was referred to our center for further assessment and treatment.

**Fig. 6:**
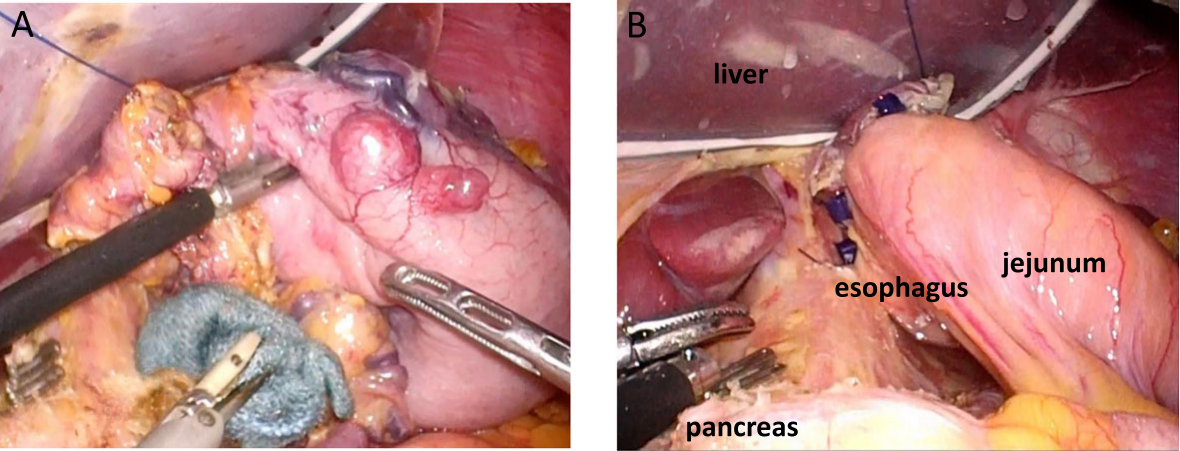
**a** Numerous nodules were observed on the serous surface of the stomach, with mild adhesions around the gastroduodenal anastomosis. **b** Completion gastrectomy and D1 plus lymph node dissection were performed laparoscopically, with a Roux-en-Y reconstruction

**Fig. 7:**
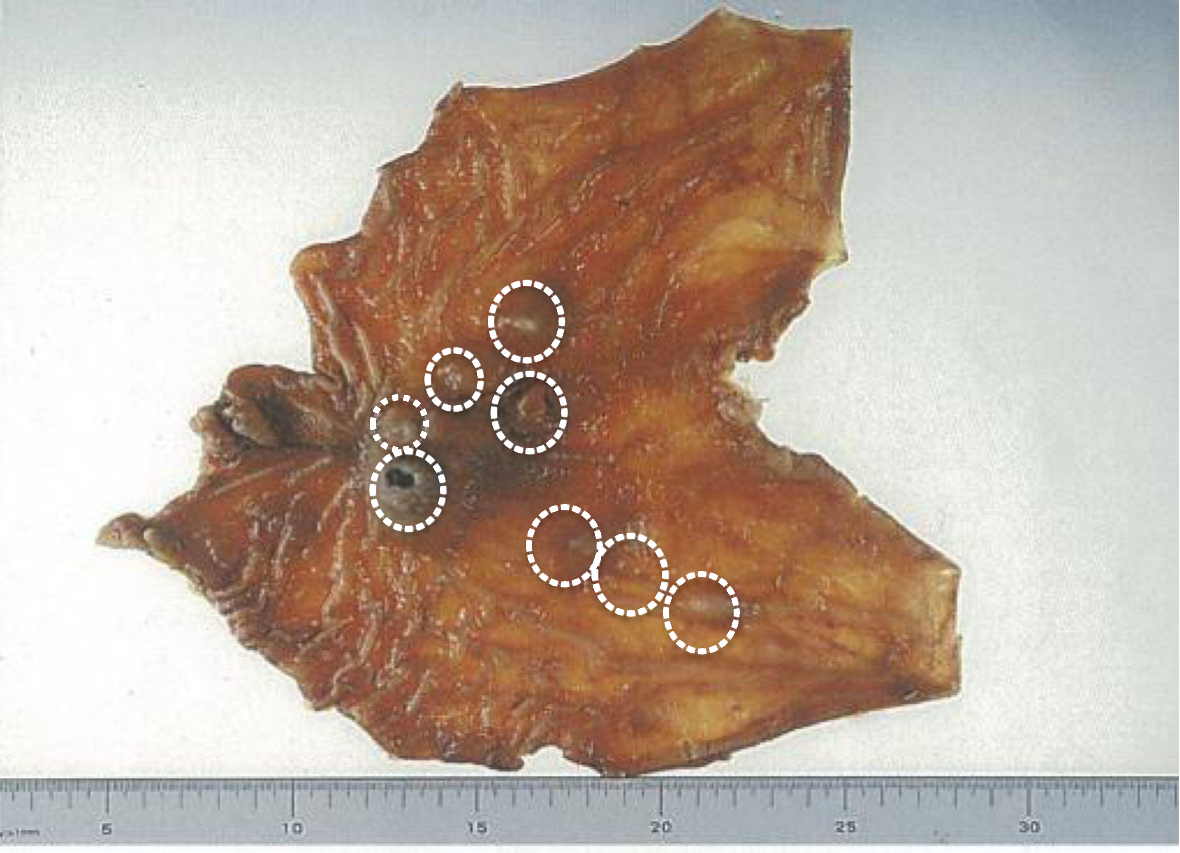
Sporadic gastric stromal tumorlets were observed, identified as multifocal subserosal polypoid nodules (*n* = 8), with the largest of these tumors being 52 mm × 30 mm × 25 mm in size

**Fig. 8:**
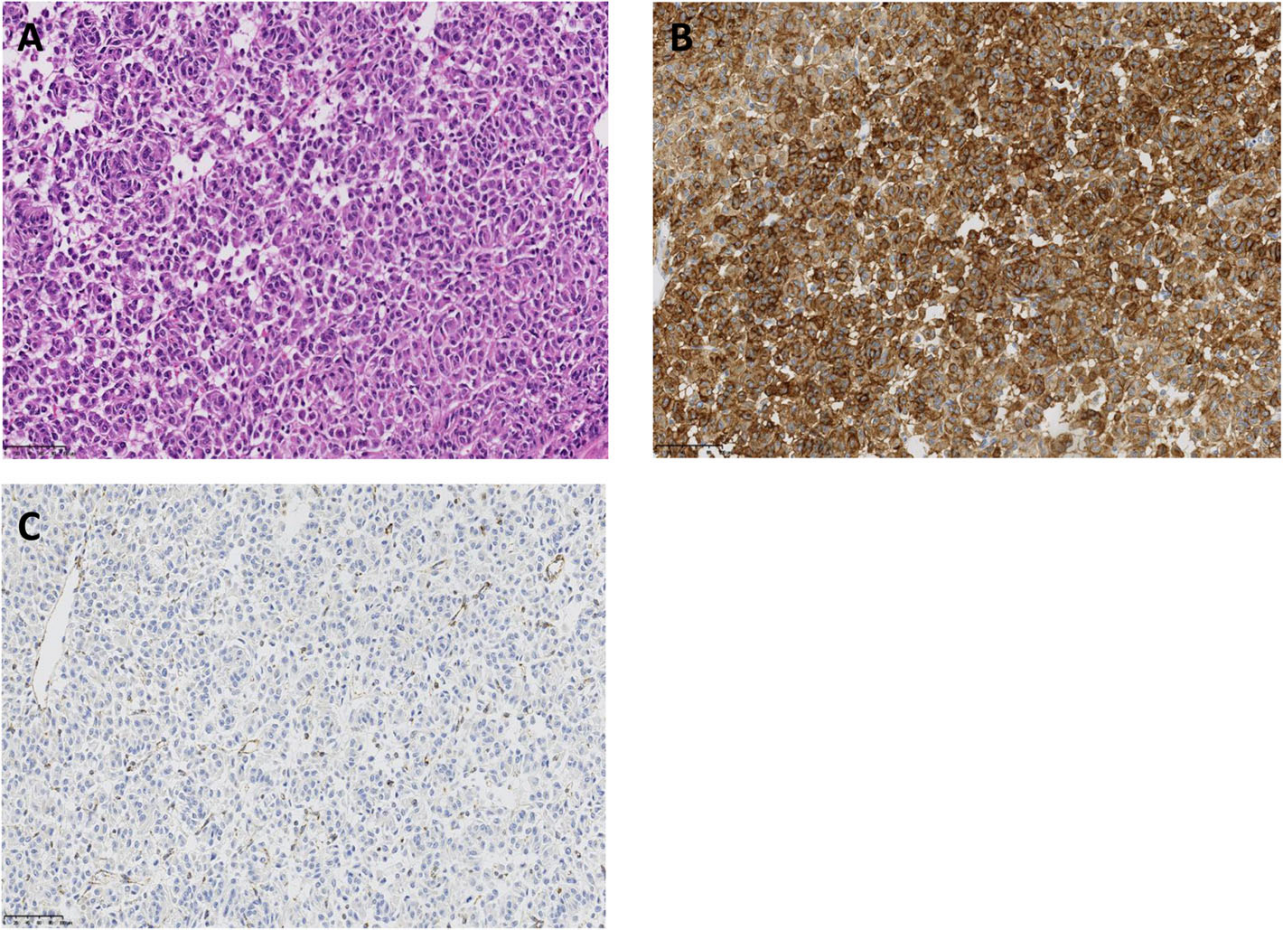
**a** Tumor cells showing an epithelioid pattern, with **b** all eight tumors being diffusely immunoreactive with KIT antibodies. **c** Tumors were succinate dehydrogenase deficient

There was no evidence of extra-adrenal paraganglioma. A clinical diagnosis of recurrent GISTs, as a component of an incomplete-type Carney’s triad, was made. We decided to proceed with laparoscopic examination and treatment, as appropriate. On laparoscopic examination, further multiple nodules were observed on the serous surface of her stomach, with mild adhesion around the gastroduodenal anastomosis that could be easily divided (Fig. 6a). Based on these findings, we proceeded with completion gastrectomy, with D1 plus lymph node dissection and Roux-Y reconstruction, performed laparoscopically (Fig. 6b). Macroscopic examination revealed that the sporadic gastric stromal tumorlets were multifocal subserosal polypoid nodules (*n* = 8), with the largest being 52 × 30 × 25 mm in size (Fig. 7). The tumor cells showed an epithelioid pattern, and all eight tumors were diffusely immunoreactive on CD117 and CD34 antibody assay (Fig. 8a–c). Vessel or lymphatic invasion was not observed. The Ki-67 index was 10%. Tumors were SDH deficient, confirming the diagnosis of GISTs as a component of an incomplete-type Carney’s triad. We did not observe any benefit of chemotherapy on the harvested tumor specimen.

There was no complication after surgery, and the patient’s postoperative course was uneventful. She was discharged from the hospital in good condition and has been monitored carefully in follow-up, with no adjuvant chemotherapy treatment. Over the 7 months since the gastrectomy procedure, she has had no difficulty with oral intake, and there is no evidence of tumor recurrence.

## Discussion

In our case, tumor recurrence occurred at a latency of 11 years after the index gastric surgery; this is a relatively longer latency period than is previously reported [3]. Regarding the surgical approach for GIST resection, an open approach was the approach of choice in previous reports [5, 10–13]. An open approach definitely renders the dissection of adhesions and the completion gastrectomy easier to perform. However, considering that partial resection is generally the treatment of choice for primary Carney-type GISTs, the risk of adhesions at the surgical site is considered to be low overall. Moreover, as Carney’s triad typically occurs in young individuals, a laparoscopic approach would be indicated cosmetically if it can be safely performed. In fact, in our case, adhesions were mild and the completion gastrectomy was relatively easy to perform with laparoscopy.

Current guidelines for the treatment of GISTs in Japan do not recommend lymph node dissection [14]. However, differences between typical GISTs and GISTs with Carney’s triad need to be considered. Carney-type GISTs grow slowly and are prone to distant metastases, including the regional lymph nodes [3, 6, 15]. Our review of the literature identified 6 previous reports in which Carney-type GISTs were treated using gastrectomy and lymph node dissection [10–13, 16, 17]; however, the lymph node status and station number were not clearly stated. In one report, which was studied in 104 patients with Carney’s triad at the Mayo Clinic, a 29% rate of lymph node metastasis has been reported for Carney-type GISTs [17]. Therefore, despite low evidence of necessity in lymph node dissection of Carney’s triad, we recommend at least D1 plus lymph node dissection for Carney’s triad.

Alternative therapies have previously been reported for the treatment of metastatic Carney-type GISTs [5]. However, chemotherapy, radiation, and thermoablation or cryoablation have been shown to be ineffective to treat these metastatic lesions [7]. Our patient had been treated with three different types of TKIs, all of which were ineffective. If the SDH deficiency of the GISTs had been confirmed at the time of first treatment, the side effect of TKI treatment could have been avoided for our patient.

## Conclusions

In summary, we report on the recurrence of GISTs in a case of incomplete-type Carney’s triad. The novel information presented herein is the successful performance of a completion gastrectomy and D1 plus lymph node dissection with laparoscopy. The postoperative course was uneventful.

## Data Availability

None.

## Abbreviations

GIST: Gastrointestinal stromal tumors
SDH: Succinate dehydrogenase deficiency
TKI: Tyrosine kinase inhibitor
